# Human alkaline phosphatase dephosphorylates microbial products and is elevated in preterm neonates with a history of late-onset sepsis

**DOI:** 10.1371/journal.pone.0175936

**Published:** 2017-04-27

**Authors:** Matthew Pettengill, Juan D. Matute, Megan Tresenriter, Julie Hibbert, David Burgner, Peter Richmond, José Luis Millán, Al Ozonoff, Tobias Strunk, Andrew Currie, Ofer Levy

**Affiliations:** 1 Precision Vaccines Program, Division of Infectious Diseases, Boston Children’s Hospital, Boston, Massachusetts, United States of America; 2 Harvard Medical School, Boston, Massachusetts, United States of America; 3 University of California Davis School of Medicine, Davis, California, United States of America; 4 The University of Western Australia, Crawley, Western Australia, Australia; 5 Murdoch Children’s Research Institute, Parkville, Victoria, Australia; 6 Department of Paediatrics, University of Melbourne, Parkville, Victoria, Australia; 7 Department of Paediatrics, Monash University, Clayton, Victoria, Australia; 8 Sanford Children's Health Research Center, Sanford-Burnham Medical Research Institute, LaJolla, California, United States of America; 9 School of Veterinary & Life Sciences, Murdoch University, Murdoch, Western Australia, Australia; Hopital Robert Debre, FRANCE

## Abstract

**Background:**

A host defense function for Alkaline phosphatases (ALPs) is suggested by the contribution of intestinal ALP to detoxifying bacterial lipopolysaccharide (endotoxin) in animal models *in vivo* and the elevation of ALP activity following treatment of human cells with inflammatory stimuli *in vitro*. However the activity of ALP in human plasma (primarily tissue-nonspecific ALP; TNAP) on lipopolysaccharide and other microbial products has not been assessed, nor has its expression been studied in preterm newborns, a vulnerable population at high risk of sepsis. In this context, the aim of our study was to characterize the activity of TNAP on *Toll*-like receptor (TLR) agonists and assess the concentrations of plasma ALP during late-onset sepsis in preterm newborns.

**Methods:**

Recombinant human TNAP was incubated with microbial products and phosphate release was measured by malachite green assay. Plasma ALP activity was measured serially in a cohort of preterm (N = 129) infants at high risk of late-onset sepsis (LOS).

**Results:**

TNAP dephosphorylates poly-inosine:cytosine (Toll-like receptor (TLR) 3 agonist) and LPS from *Klebsiella pneumoniae* and *Salmonella minnesota* (TLR4 agonists). Plasma ALP significantly increased postnatally over the first 4 weeks of life in preterm and term newborns. Bacteremic LOS in preterm infants (gestational age ≤ 30 weeks) was associated with significantly elevated plasma ALP at 4 weeks postnatal age.

**Conclusions:**

TNAP, the main circulating isozyme of ALP, de-phosphorylates TLR agonists, demonstrates a post-natal age dependent increase in preterm and term plasma across the first 4 weeks of life, and is elevated in association with preterm LOS.

## Introduction

Newborn infants, especially those born preterm, are at increased risk of infection [1, 2], and their ability to control inflammation during microbial colonization and infection during early life is critical to proper development and homeostasis [3, 4]. Alkaline phosphatase (ALP) contributes to the regulation of inflammation and protects experimental animals from septic challenge. There are 4 distinct human ALP genes: *ALPL* (tissue-nonspecific ALP, or TNAP, the predominant circulating form, with high levels in bone, kidney, and liver), *ALPP* (placental ALP), *ALPPL2* (germ cell ALP), and *ALPI* (intestinal ALP). Intestinal ALP dephosphorylates lipopolysaccharide (LPS, endotoxin) [5, 6]. Zebrafish intestinal ALP protects from inflammation and mortality due to LPS stimulation [7], and murine intestinal ALP protects gut barrier function and the normal homeostasis of the gut microbiota [8, 9].

A variety of inflammatory stimuli induce ALP expression. Interleukin (IL)-6, an important mediator of the acute phase response, induces up-regulation of ALP on endothelial cells [10]. In rodents, pathogen associated molecular patterns (PAMPs) such as LPS induce ALP in kidney glomerular and endothelial cells [11] and liver tissue [12], and uterine ALP promotes implantation, decidualization, and contributes to defense against bacterial LPS [13]. In human phagocytes, LPS induces ALP activity *in vitro* [14]. Exogenous ALP may have potential therapeutic applications. Intestinal ALP reduces the inflammatory capacity of LPS via dephosphorylation of exposed phosphate groups [6, 9], and also influences microbial growth in the gut [9, 15, 16]. Calf intestinal alkaline phosphatase (cIAP) is an effective therapeutic agent, protecting mice from lethal *E*. *coli* challenge, and reducing tumor necrosis factor α (TNFα) levels in piglets subsequently injected with LPS [17]. Exogenous intestinal ALP (cIAP) has been evaluated in adult human clinical trials to evaluate safety and clinical pharmacology [18], and further assessed for efficacy in improving renal function during Gram-negative sepsis in a randomized double-blind, placebo-controlled study [19]. Intravenous infusion of ALP, started within 48 hours of acute kidney injury, increased endogenous creatinine clearance (baseline to Day 28) relative to controls, and significantly reduced systemic and urinary inflammatory biomarkers [19]. Alkaline phosphatase also was demonstrated to protect from renal inflammation by dephosphorylating LPS and ATP in an animal model [20]. Of note, human plasma ALP, which is higher in newborns than adults, may also reduce inflammation via enzymatically converting adenine nucleotides (ATP, ADP, and AMP) to adenosine, an endogenous purine metabolite that acts via cognate seven-transmembrane receptors to induce cyclic AMP, thereby inhibiting inflammatory processes such as neutrophil migration [21] and ROS generation [22] and leukocyte production of pro-inflammatory cytokines [23].

Toll-like receptor (TLR) stimulation leads to induction of ALP expression in a variety of tissues and cell types [8], and ALP dephosphorylates LPS and possibly other TLR agonists (TLRAs). We therefore assessed whether human TNAP would dephosphorylate TLRAs. In addition we sought to define the ontogeny of plasma ALP in preterm and term infants, and the relationship with bacteremic late-onset sepsis (LOS) in a prospective cohort of preterm infants. In order to determine if exposure to chorioamnionitis (HCA), and/or development of LOS, modulated circulating levels of ALP in plasma, we sought to evaluate serially collected plasma samples for total ALP activity, and performed statistical analysis with grouping based on clinical evaluation of subjects. We find that human TNAP dephosphorylates LPS and the double-stranded RNA (dsRNA, TLR3 agonist) mimic pI:C. Infants who developed bacteremic LOS had significantly elevated plasma ALP at 4 weeks postnatal age, compared to infants without LOS. Our findings provide fresh insight into the biology and regulation of circulating human ALP and highlight its potential as a protein therapeutic in neonatal sepsis.

## Methods

### Ethical approval and study design

The King Edward Memorial Hospital (Perth, Australia) Ethics Committee approved the study protocol and written, informed consent was obtained from the parents of subjects. Cord or peripheral blood was collected from very preterm patients (<30 weeks GA, N = 129 included), and a cohort of healthy term patients as a comparison arm (>37 weeks GA, N = 20 included). Histological chorioamnionitis (HCA) was evaluated in the preterm infants by standardized histology of placentae as previously described [24]. Histology data was available for 112 of the 129 preterm infants in the study. Late-onset neonatal sepsis was defined by isolation of a single organism from blood culture (>72 hours after delivery) and on the level of circulating C-reactive protein (CRP) and divided into the following groups: No LOS (no signs or symptoms of LOS and not tested, or culture negative and CRP<15 mmol/l), contamination (culture positive with microorganism commonly causing contamination (coagulase negative *Staphylococcus*, diphtheroids, etc.) and CRP<15), possible LOS (culture negative and CRP>15), and definite LOS (positive culture and CRP>15). Four subjects developed early-onset sepsis (EOS, <72 hours after delivery), and due to limited numbers of subjects they were excluded from further analysis.

### Blood collection

Umbilical cord blood was collected into pre-heparinized syringes as soon as possible postnatally from cord vessels, and where necessary, from vessels feeding into cord vessels, immediately adjacent to the base of the cord on the fetal placental surface. Preterm peripheral blood was collected by venipuncture (or from central arterial catheters if available; D1 only) on Days 1 (median age 10 hours, range 0.55 to 23.47 hours), 7 (median age 7.02 days, range 6.21 to 8.34 days), 14 (median age 14.04 days, range 12.66 to 15.30 days), 21 (median age 21.01 days, range 19.72 to 23.14 days) and 28 (median age 28.03 days, range 26.35 to 30.11 days) into lithium-heparin tubes (Becton Dickinson). Term peripheral blood was collected by venipuncture on days 1 (median age 5 hrs) and 28 (median age 28.13 days) into lithium-heparin tubes. All blood samples were collected into tubes containing Erythro-9-(2-Hydroxy-3-nonyl)adenine hydrochloride (EHNA, an inhibitor of adenosine deaminase) and 2,6-bis(Diethanolamino)-4,8-dipiperidinopyrimido[5,4-d]pyrimidine (dipyridamole, an inhibitor of nucleoside transporters) for parallel studies on purine levels. Neither dipyridamole nor EHNA influence ALP activity (data not shown). Blood samples were centrifuged at 6,000 x g for 2 minutes at room temperature, and plasma was removed and stored at -80°C. Blood sampling was not performed from each subject at each time-point, but a total of 542 plasma samples were collected from 129 pre-term subjects, and a total of 44 plasma samples collected from 20 healthy term subjects (See Table 1).

**Table 1:** 

	Pre-term						Term
	Total	EOS	Non-LOS	Possible LOS	Definite LOS	HCA	Total
# Subjects	129	4	78	18	29	53	20
# Female	59	2	34	10	13	28	11
C-section	73	2	44	11	16	21	3
GA Range	22.85–29.85	23.42–28.42	23.28–29.85	22.85–29.57	23.28–29.85	22.85–29.85	38.1–42.00
GA Median	27.14	25.21	27.71	25.57	26.57	26.71	40.14
GA Mean	27.02	25.56	27.47	26.18	26.55	26.55	39.99
BW Range	455–1565	635–1295	490–1565	455–1250	510–1280	490–1510	2360–4610
BW Median	895.00	845.00	1000.00	740.00	825.00	960.00	3544.00
BW Mean	942.82	905.00	1014.74	784.11	853.10	961.72	3464.90

### Phosphate release from TLR agonists mediated by human TNAP

Recombinant human tissue-nonspecific ALP (rTNAP, R&D Systems, Minneapolis; MN, USA) was diluted 1:1000 from manufacturer’s stock (0.3 mg/mL) in assay buffer (50 mM Ca^2+^ and Mg^2+^, 20 μM Zn^2+^, and 40 mM Tris). TLR agonists were prepared in molecular biology grade water (Invitrogen) to a final concentration of 400 μg/mL unless otherwise indicated: synthetic diacylated lipoprotein FSL-1 (40 μg/mL), low molecular weight Poly(I:C) (4 mg/mL), monophosphoryl lipid A from *Salmonella minnesota* Re595 (MPLA-SM), R848 (750 μM), CpG (ODN2216), endotoxin-free *Escherichia coli* DNA (EcDNA, Invivogen), *Salmonella minnesota* Re 595 LPS (LIST Biochemical), *Klebsiella pneumonia* LPS (Sigma), and adenosine mono-phosphate (AMP, Sigma). 66 μL enzyme combined with 44 μL buffer, AMP control, or TLR agonist, and incubated at 37°C in a heat block for 30 minutes. 50 μL (duplicates) of mixture evaluated for free inorganic phosphate (Pi) by Malachite Green Assay (R&D Systems, Minneapolis; MN, USA) per the manufacturer’s instructions. TLRA and control reagents with buffer were also evaluated for Pi concentration with each assay. Malachite Green Assay Buffer A caused precipitation of *Salmonella minnesota* Re LPS, CpG, and pI:C, which were centrifuged (1000g, 2 minutes) and the supernatant used for further analysis. There was a modest amount of Pi in the enzyme preparation and in some of the TLRA reagents, thus relative comparisons were made plotted as [Pi (buffer + TLRA)] and [Pi (TNAP + TLRA reaction)]. Statistical comparison was made between [Pi (TNAP + TLRA reaction)–Pi (buffer + TLRA)] and [Pi (buffer + TNAP)–Pi (buffer alone)].

### Plasma ALP measurement

Total plasma ALP activity was evaluated in 1 M DEA buffer (Sigma) with 1mM Mg^2+^, 20μM Zn^2+^ and 2.7 mM p-nitrophenyl phosphate (p-NPP, Sigma) at pH 9.8, at room temperature [25]. 195 μL of buffer was added to 5 μL of sample in a 96-well plate (Corning), and absorbance at 405 nm evaluated at 5, 10, and 15 minutes after reaction initiation with a VersaMax Microplate Reader (Molecular Devices; Sunnyvale, CA, USA). p-NPP dephosphorylation product p-NP was compared to serially diluted p-NP controls of known concentrations, and the rate of p-NP generation was calculated for each interval and averaged. ALP activity was calculated as U/L, which is μmol p-NP generated/minute/liter of plasma (μmol/min/L). Samples were evaluated in duplicate. For inhibition assays, 30 μM MLS-0038949 (EMD Millipore; Billerica MA, USA) or 10 mM L-phenylalanine (L-Phe, VWR; Radnor PA, USA) were added to appropriate wells, and the assay was run as indicated above, but with 175 μL of buffer added to 5 μL of sample, and 20 μL of inhibitor or vehicle (indicated inhibitor concentrations are final concentrations).

Statistical analysis was performed using Prism 5 (version 5.09) for Mac OS X (Graphpad Software, Inc.; La Jolla, CA, USA) and the R statistical system v3.1.0 (R Institute, Vienna Austria). Hypothesis tests are two-sided with 0.05 significance level, and p-values are Bonferroni-adjusted for multiple comparisons. Between-group comparisons use t-test or ANOVA as noted. For longitudinal models, mean values between time-points are compared using linear regression fit with generalized estimating equation (GEE) to account for repeated measures. Models controlled for sex, gestational age, and birthweight. Calculated p-values are Bonferroni-adjusted for multiple comparisons.

## Results

### The predominant alkaline phosphatase in human plasma, TNAP, de-phosphorylates TLR agonists

**Fig 1:**
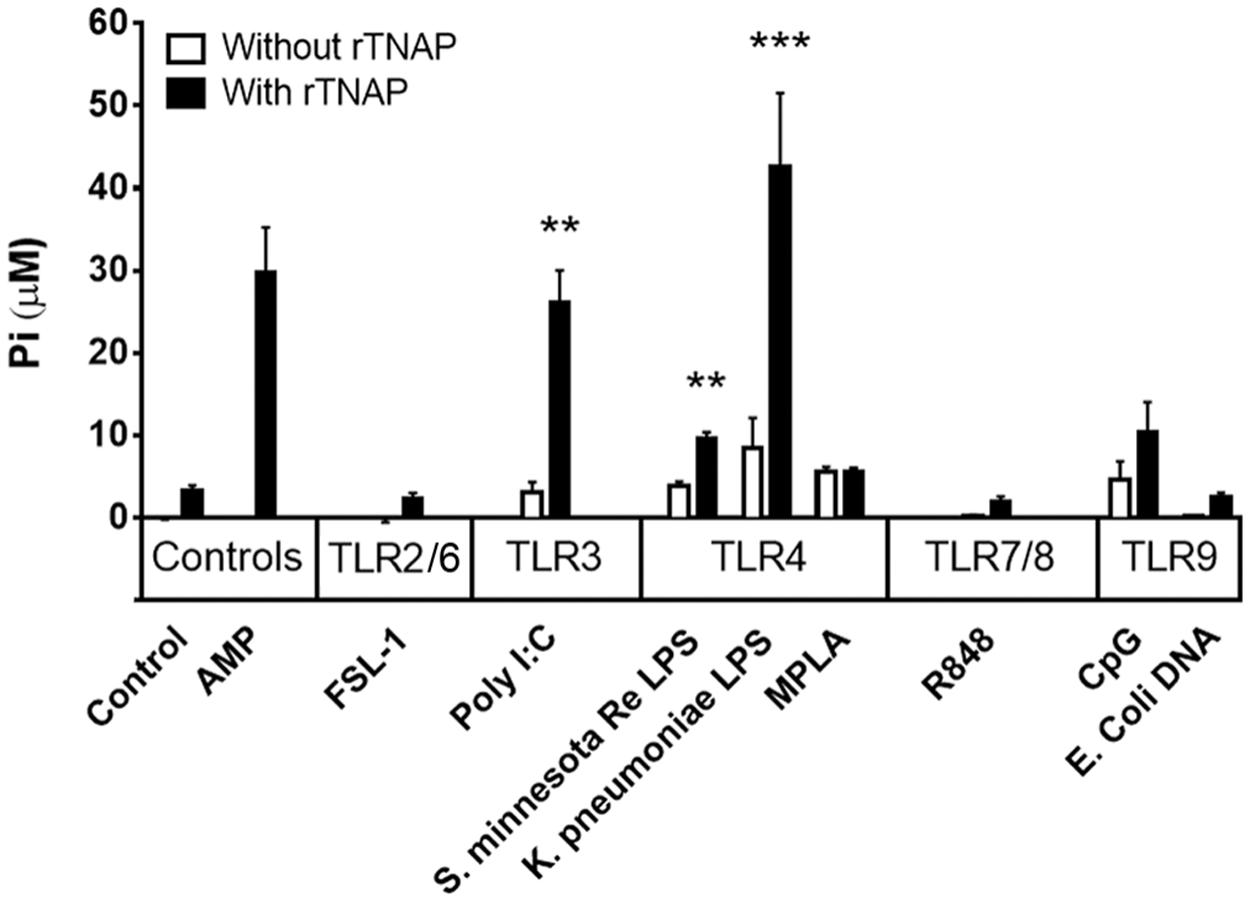
Human TNAP, the predominant AP of human plasma, dephosphorylates select TLR3 and TLR4 agonists. N = 3–7, each TLRA evaluated in at least 3 independent experiments, two-tailed Student’s t-tests. ** p<0.01, *** p<0.001.

The predominant form of ALP in blood plasma of newborn and adult humans is TNAP [23]. While it has been demonstrated that other forms of ALP, in particular intestinal ALP (IAP), dephosphorylates LPS, we sought to evaluate the ability of human TNAP to dephosphorylate LPS and other TLRAs. Recombinant human TNAP was incubated independently with and without several different TLRAs, subsequent release of inorganic phosphate (Pi) was measured by Malachite Green Assay, and data shown are adjusted to Pi detected in reagent controls as described in methods. Activity of the enzyme was confirmed for each assay by observing complete dephosphorylation of 20 μM adenosine monophosphate (AMP), and R848, a TLR7/8-activating imidazoquinoline compound that does not have a phosphate group, was included as a negative control. Human TNAP significantly dephosphorylated the double-stranded RNA mimic Poly I:C (p = 0.002, TLR3A) as well as LPS from *Klebsiella pneumonia* (p = 0.0006) and *Salmonella minnesota* Re595 (p = 0.009, TLR4As; Fig 1). Thus, human TNAP can dephosphorylate LPS (endotoxin), and other select TLRAs with exposed phosphate groups (pI:C).

### ALP activity increases in preterm neonatal plasma during the first 2 weeks of life

**Fig 2:**
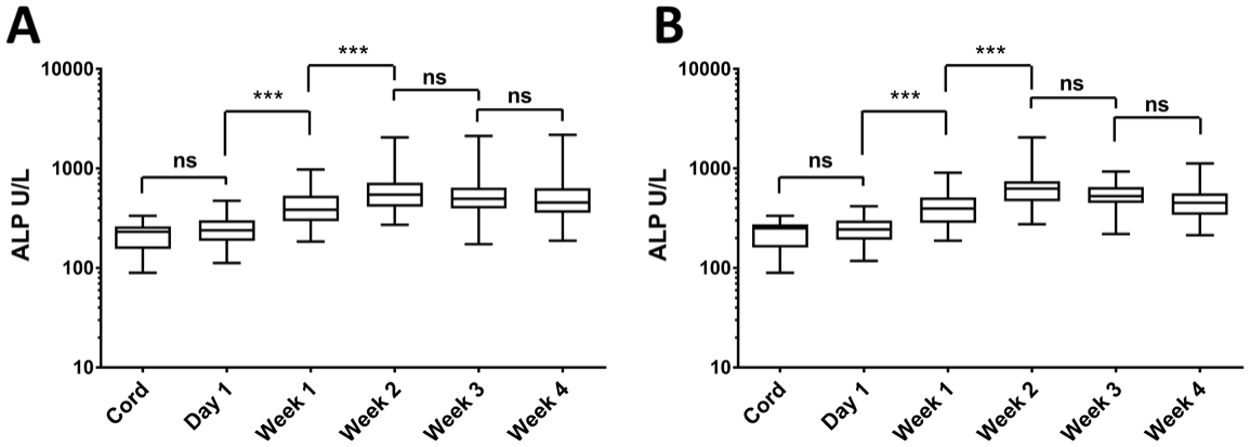
Ontogeny of plasma ALP in the first 4 weeks of life for pre-term newborns. Significance tests use repeated measures linear regression. Figure shows boxplots for all pre-term samples (A) or for only pre-term subjects without HCA or LOS (B). ** p<0.01, and *** p<0.001.

**Fig 3:**
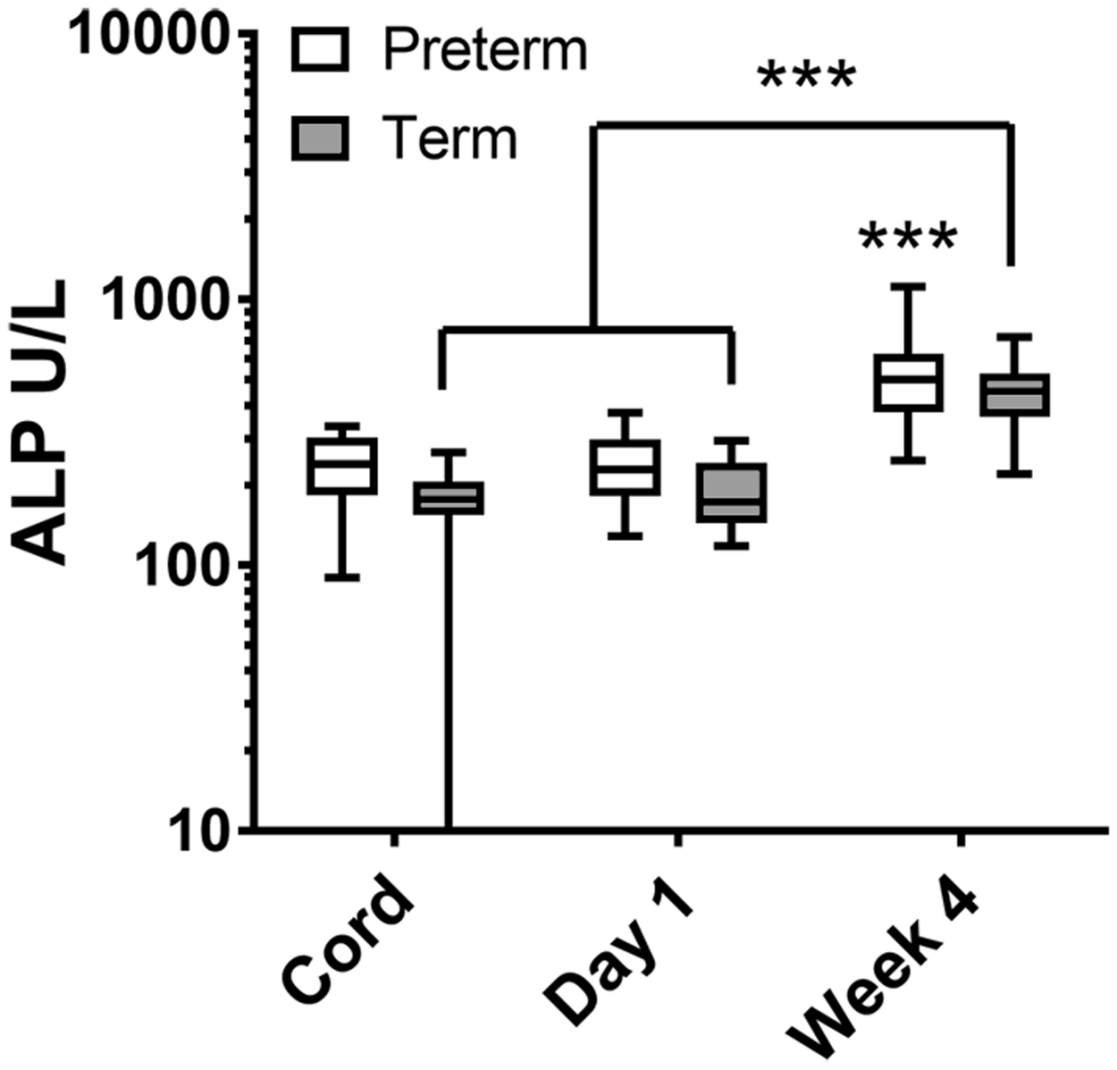
Plasma ALP increases significantly in the first 4 weeks of life for both pre-term and term newborns. Comparisons were made by ANOVA with Bonferroni multiple comparison correction (statistical results for comparison to early time points in both populations). Only subjects without HCA or LOS were included, N = 11–16 for term, N = 12–36 for pre-term. *** p<0.001.

**Fig 4:**
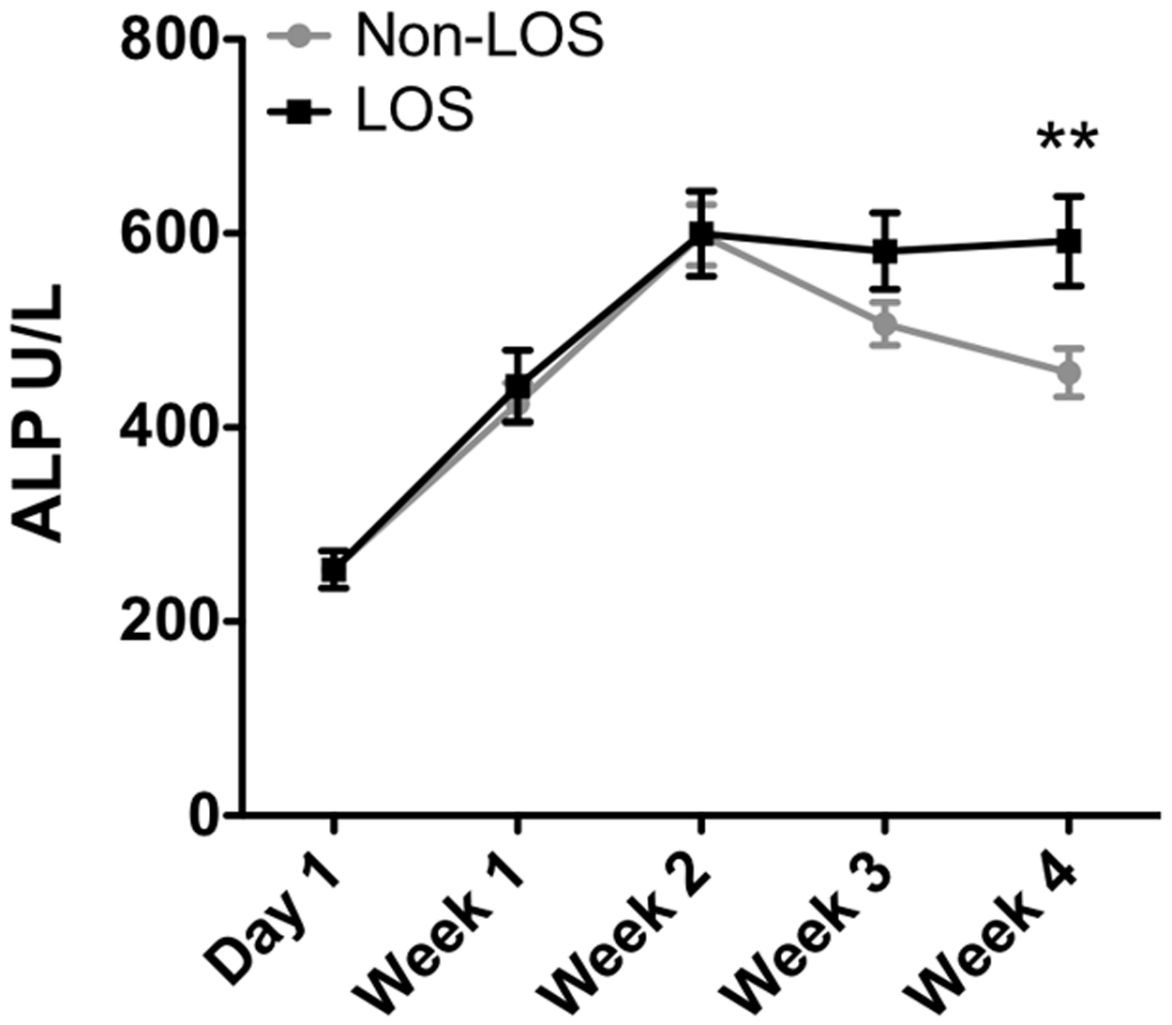
Late-onset sepsis (LOS) leads to significantly increased plasma AP in pre-term newborns. Comparison by one-way ANOVA with Bonferroni multiple comparison correction of log-transformed data at weeks 2–4, LOS N = 25,24,24 and non-LOS N = 67,54,63 at 2, 3, and 4 weeks post-delivery respectively. ** p<0.01.

Significant changes in the levels of circulating ALP were observed during the first weeks of life in both preterm (Figs 2 and 3) and term infants (Fig 3). ALP activity more than doubled in the first 2 weeks of life in preterm infants, peaked at 2 weeks postnatal age, then diminished slightly through week 4. Although blood samples were not collected at weeks 1–3 from term infants ALP levels at 4 weeks postnatal age were significantly higher than at birth (Fig 3). HCA exposure was not associated with any modulation of circulating levels of ALP at birth or at early time-points (S1 Fig). There were modest differences (lower ALP in HCA subjects) at later time-points that did not reach statistical significance (HCA vs Non-HCA, week 2 p = 0.26; week 3, p = 0.24; week 4, p = 0.59, analysis as described for Fig 4)

### Late onset sepsis is associated with significant elevation of plasma ALP

Subjects were categorized as No LOS (culture negative and CRP<15 mmol/l, 78 subjects), suspected contamination (culture positive and CRP<15, 0 subjects), possible LOS (culture negative and CRP>15, 18 subjects), and definite LOS (positive culture and CRP>15, 29 subjects). While early-onset sepsis (EOS) is also a major contributor to neonatal morbidity and mortality [26, 27], too few subjects in this cohort developed EOS (N = 4) for further analysis. The majority of the subjects in this study with definite LOS were infected with Gram-positive organisms (predominately coagulase-negative *Staphylococcus*, CoNS (20 cases), a considerable cause of invasive disease in low birth-weight neonates [28]), 2 subjects with *Enterococcus faecalis*, 1 each with *S*. *aureus* and *Bacillus sphaericus*. Additionally 5 of the 29 definite LOS cases were caused by Gram-negative bacteria (3 cases with *Escherichia coli*, and 1 each *Enterobacter cloacae* and *Klebsiella pneumoniae*). The first positive culture occurred with a range from day of life (DOL) 6 of life to DOL26, with the majority of definite LOS subjects having a first positive culture between DOL10-18. Development of LOS was associated with significantly elevated plasma ALP at 4 weeks postnatal age (Fig 4). A similar association was observed with possible LOS (S2 Fig). As infants developing definite LOS were more likely to have lower GA and birth weight (Table 1) than Non-LOS subjects, we evaluated ALP values at DOL1 and week 1, and also the changes from birth to week 1 (p = 0.9937, Spearman test, data not shown), and found no significant differences in ALP levels in these populations at early time points (Fig 4).

MLS-0038949 (30 μM), a potent and specific inhibitor of TNAP [29], blocked ~ 90% of total plasma ALP activity in samples at 4 weeks post-natal age regardless of LOS status, plotted as fraction of remaining activity compared to vehicle controls for the same subjects (S3A Fig). L-phenylalanine (L-Phe, 10 mM), a partial inhibitor of IAP, PLAP, and GCAP, but a weak inhibitor of TNAP, inhibited ~25% of total plasma ALP in both populations (S3B Fig). The pharmacologic inhibition profile indicates that plasma ALP activity at 4 weeks post-natal age is primarily due to TNAP in both populations.

## Discussion

Preterm infants are particularly susceptible to infection and inflammatory sequelae. ALP regulates inflammation in the gut by dephosphorylating microbial molecules, and may contribute to control of excessive inflammation. Intestinal ALP has been shown to be useful for the treatment of sepsis in animal models [17], and in humans [19]. Inflammatory stimuli may induce tissue-nonspecific ALP (TNAP) *in vitro* [11, 12, 14]. Sepsis induces elevation of ALP in adult patients [30, 31]. It was not previously known if circulating levels of ALP were elevated during or after sepsis in neonates. Increased production of ALP could comprise a host strategy to alleviate excessive inflammation during systemic exposure to microbial products. Here we demonstrate that endogenous plasma ALP (predominantly TNAP) is modestly, but significantly, elevated in preterm neonates who developed LOS (Fig 4).

Bovine intestinal ALP dephosphorylation of LPS from Gram-negative bacteria leads to diminished subsequent inflammatory responses [5, 6, 32], and there are likely multiple foreign molecules for which ALP-mediated dephosphorylation, modulates host receptor interactions. In a human clinical sepsis trial [19] that excluded patients with Gram-positive and fungal culture positive infections, exogenous ALP improved renal function during sepsis whether the patients had confirmed Gram-negative sepsis or not. While the molecular biology of sepsis is complex [33], these ALP mediated improvements in renal function could reflect culture-negative endotoxemia [34], potential effects of ALP in detoxifying microbial products other than LPS, and/or alteration by ALP of host responses via production of anti-inflammatory adenosine by dephosphorylating extracellular adenosine monophosphate (AMP) [23].

Bovine intestinal ALP dephosphorylates LPS (TLR4A), bacterial flagellin (TLR5A), UDP and bacterial CpG DNA (TLR9A) thereby reducing their inflammatory activities [32, 35]. Serum levels of ALP are commonly measured clinically, but we demonstrate for the first time that human TNAP, the predominant circulating form of ALP, dephosphorylates LPS and other foreign molecules. We demonstrate here that human TNAP significantly dephosphorylates the double-stranded RNA mimic pI:C (TLR3A) as well as LPS from *Klebsiella pneumoniae* and *Salmonella Minnesota* (TLR4As; Fig 1). In contrast, we observed only modest increases in free phosphate following incubation of human TNAP with CpG, and did not detect phosphate release from *E*. *coli* DNA. It is likely that TNAP mediates release of terminal phosphates from microbial DNA, but that given the size of the DNA molecule the molar concentration of free phosphate generated was below the limit of detection in our assay. *In vivo*, TNAP may contribute to detoxifying bacterial DNA that is subject to attack by host DNAses potentially unmasking additional terminal phosphate sites.

We noted significant differences in plasma ALP levels in the first 4 weeks of life in both preterm and term neonates. Circulating bone ALP (an isoform of TNAP) and fetal intestinal ALP increase in the first weeks of life in both preterm and term neonates [8, 13]. In particular, fetal intestinal ALP was higher in preterm neonates and, although still constituting a minority of total AP activity compared to bone ALP, is highest at lower gestational age. The ontogeny of total plasma ALP activity was not evaluated in these populations. A trend for elevated intestinal ALP in term infants at birth who subsequently developed necrotizing enterocolitis, compared to those who did not, was noted, but the limited number of study subjects precluded a definitive conclusion [8]. In our study, ALP levels more than doubled in the first 2 weeks of life in preterm neonates. Although the 2-week time point was not available for term neonates, they also displayed significantly increased ALP at 4 weeks post-natal age to similar levels as observed in preterm neonates. Our studies regarding the changes in post-natal plasma ALP activity in premature infants <30 weeks gestational age may prove a useful reference as clinicians consider plasma ALP levels while evaluating renal or liver function in these populations.

Overall, our study features multiple strengths including the first biochemical demonstration of the ability of human TNAP, the main circulating ALP, to de-phosphorylate TLRAs such as LPS and poly I:C, the first description of plasma ALP ontogeny in a cohort of preterm and term human newborns and the first evaluation of the impact of LOS on ALP levels.

Our study also has important limitations. We tested ALP activity against TLRAs *in vitro* but its contribution to host defense *in vivo* is likely impacted by other soluble and cellular host defense systems. With respect to our clinical cohort, subjects with LOS likely received different medications than the Non-LOS group, some of which may contribute to liver or kidney stress and elevated ALP, potentially confounding interpretation. Additionally, ALP was measured in samples collected at predetermined times (DOL), and not at dates of collection of blood for blood culture. Only 6 of the 29 LOS subjects had an ALP measurement within 24 hours of blood collected resulting in a positive culture, and while 5 of the 6 had ALP measurements elevated relative to the Non-LOS mean for the same collection time. Thus the limited number of subjects for which ALP was measured in close temporal proximity to blood culture positivity precludes a meaningful analysis of the precise time relationship between sepsis and ALP levels.

Overall, in the context of the known function of ALP, our study suggests that increases in circulating ALP during or following sepsis may constitute part of a host response targeting excessive inflammation caused by cellular recognition of microbial PAMPs. Plasma ALP may play important anti-inflammatory roles via at least two mechanisms: (a) via dephosphorylation of ATP/ADP, contributing to generation of adenosine, an anti-inflammatory purine metabolite that acts via seven-transmembrane G-protein coupled leukocyte adenosine receptors to inhibit generation of pro-inflammatory cytokines [23] and (b) via de-phosphorylation and inactivation certain TLRAs. By these mechanisms, endogenous ALP, including TNAP, may contribute to the regulation of inflammation, and exogenous ALP may have utility as a therapeutic agent to reduce microbe-induced inflammatory responses. As the potential benefit of anti-inflammatory therapies may depend on multiple factors (eg, microbial inoculum, host genetics and epigenetics, concurrent exogenous antibiotics, etc), further studies are indicated to assess the relationship of ALP to bacterial sepsis in the preterm.

## Supporting information

S1 Fig Histologic chorioamnionitis did not significantly impact ALP concentrations. HCA vs. non-HCA compared at all time-points by ANOVA with Bonferroni multiple comparison correction, no significance, N = 41–52.
(TIF)

S2 Fig Possible LOS vs non-LOS ALP (log transformed) compared by one-way ANOVA with Bonferroni multiple comparison correction at weeks 2–4, possible LOS N = 7,12,and 16 at 2, 3, and 4 weeks post-delivery respectively, non-LOS N = 67, 54, and 63 at 2, 3, and 4 weeks post-delivery respectively. ** p<0.01.
(TIF)

S3 Fig The majority of circulating plasma ALP in both definite LOS and non-LOS subjects is TNAP. ALP activity measured in the presence of (A) MLS-0038949, a potent and selective TNAP inhibitor, or (B) L-phenylalanine, an inhibitor of IAP, PLAP, and GCAP, but a weak inhibitor of TNAP. N = 16 each group, from definite-LOS samples with sufficient volume remaining at the 4 week collection and 16 randomly selected non-LOS samples, values shown are relative to each sample’s control condition. Both inhibitors significantly reduce ALP activity, paired Student’s t-tests, *** p<0.001. Two-tailed Student’s t-tests indicate no significant differences between definite LOS and non-LOS groups for either pharmacological inhibitor.
(TIF)

S1 Data DATA FILE_Alk phos preterm sepsis chorio clinical data for Ofer Levy—VERIFIED. Excel data file including alkaline phosphatase measurements and subject clinical data.
(XLSX)

S2 Data DATA FILE_TNAP TLRa malachite green Fig 1. Excel data file including measurements for alkaline phosphatase mediated dephosphorylation of various TLR agonists and control molecules *in vitro*.
(XLSX)

## Abbreviations

ADP: Adenosine diphosphate
ALP: alkaline phosphatase
AMP: Adenosine monophosphate
ATP: Adenosine triphosphate
cIAP: Calf intestinal alkaline phosphatase
CoNS: coagulase-negative staphylococci
CpG: C-phosphate-G DNA
CRP: C-reactive protein
EcDNA: Escherichia coli DNA
FSL-1: synthetic diacylated lipoprotein
GA: gestational age
GCAP: germ-cell ALP
HCA: histologic chorioamnionitis
IAP: intestinal ALP
LOS: Late-onset sepsis
L-Phe: L-phenylalanine
LPS: lipopolysaccharide
MPLA: monophosphoryl lipid A
PAMPs: Pathogen-associated molecular pattern molecules
pI:C: poly-inosine:cytosine
PLAP: placental ALP
p-NPP: p-nitrophenyl phosphate
rTNAP: recombinant tissue-nonspecific alkaline phosphatase
TLR: Toll-like receptor
TLRa: Toll-like receptor agonist
TNAP: tissue-nonspecific alkaline phosphatase
TNFα: Tumor necrosis factor alpha
