# Correction: Human alkaline phosphatase dephosphorylates microbial products and is elevated in preterm neonates with a history of late-onset sepsis

**DOI:** 10.1371/journal.pone.0197532

**Published:** 2018-05-10

**Authors:** Matthew Pettengill, Juan D. Matute, Megan Tresenriter, Julie Hibbert, David Burgner, Peter Richmond, José Luis Millán, Al Ozonoff, Tobias Strunk, Andrew Currie, Ofer Levy

There is an error in the XML that is causing the seventh author’s name, Jose Luis Millan, to be indexed incorrectly. The name should be indexed as Millan, Jose Luis.
