# Supplementary figures and images for: Human alkaline phosphatase dephosphorylates microbial products and is elevated in preterm neonates with a history of late-onset sepsis
Source: PLoS One. 2017 Apr 27;12(4):e0175936. doi: 10.1371/journal.pone.0175936 (PMC5407836; doi:10.1371/journal.pone.0175936)

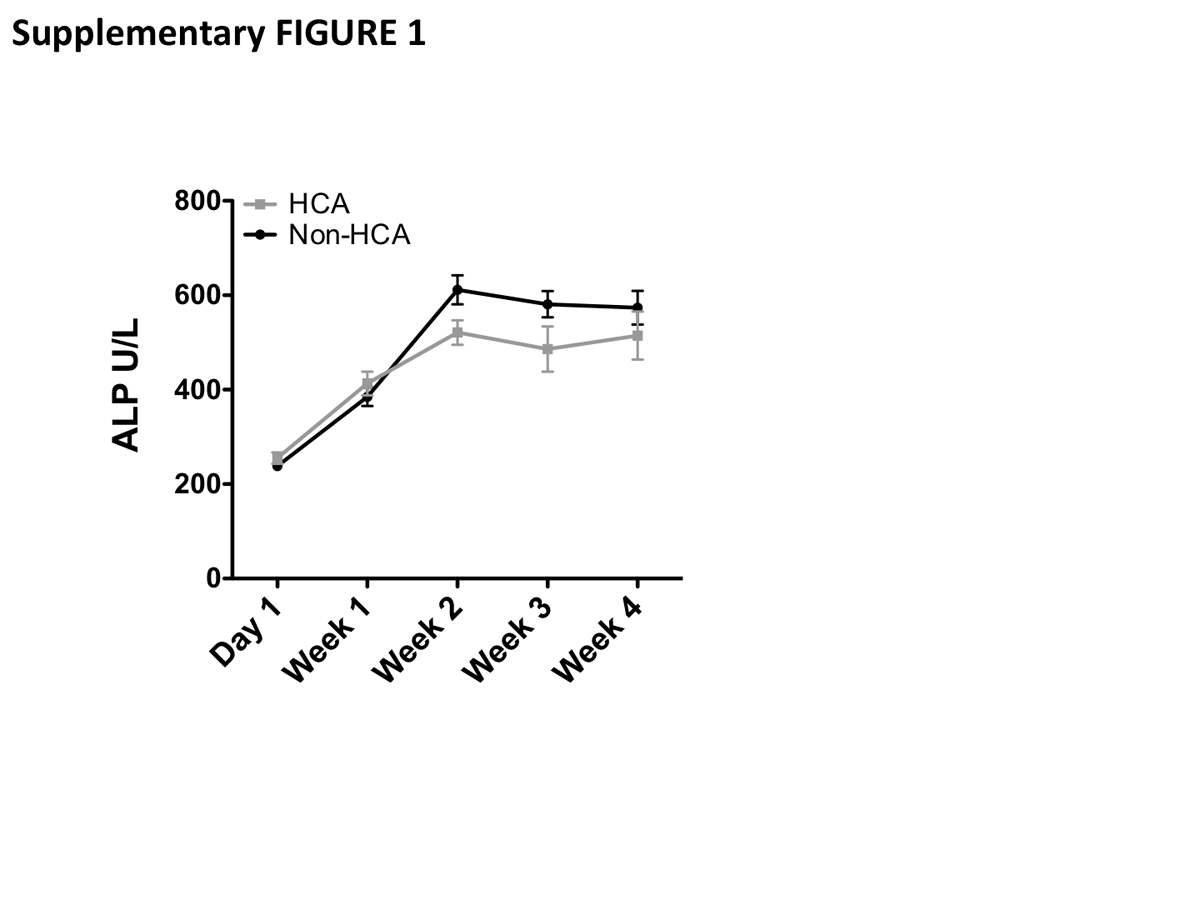

Supplement: S1 Fig — HCA vs. non-HCA compared at all time-points by ANOVA with Bonferroni multiple comparison correction, no significance, N = 41–52. (TIF) [file pone.0175936.s001.tif]

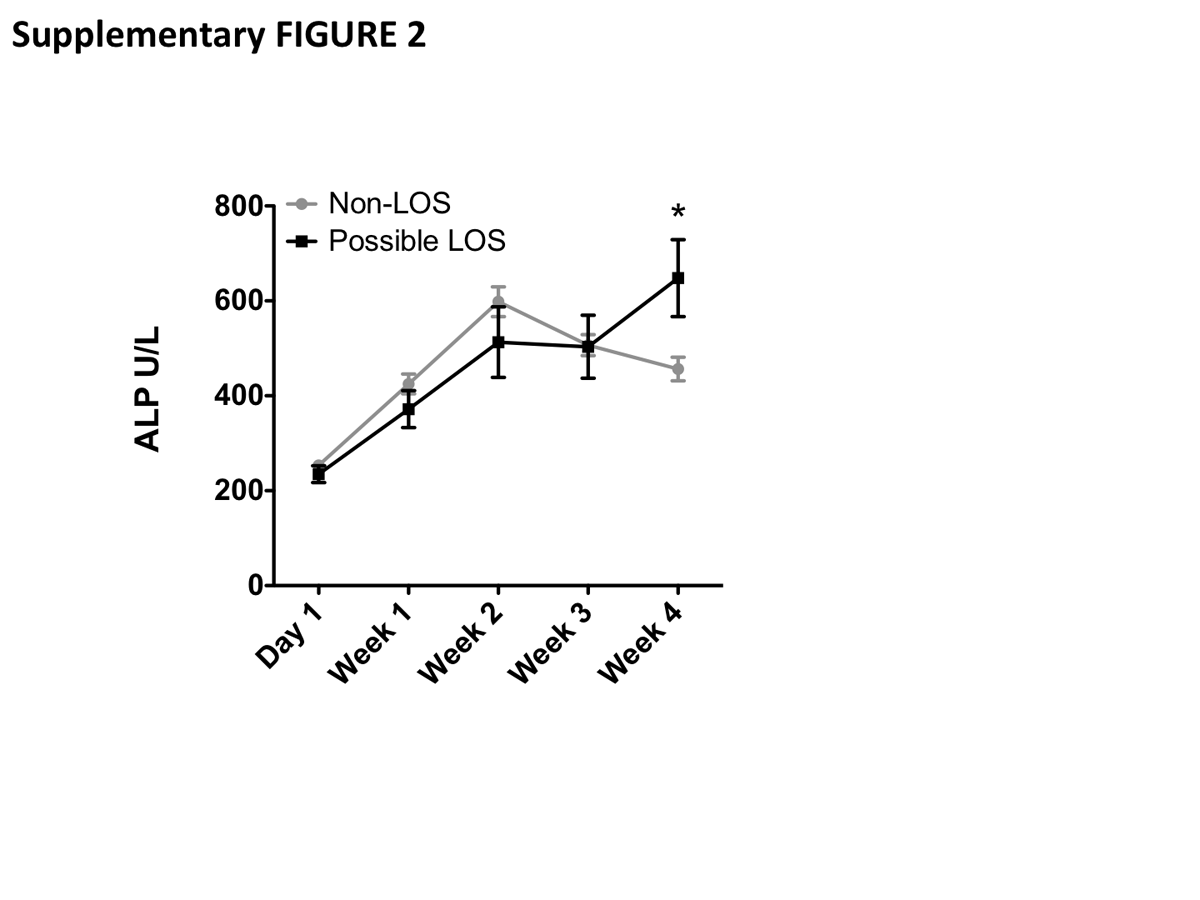

Supplement: S2 Fig — ** p<0.01. (TIF) [file pone.0175936.s002.tif]

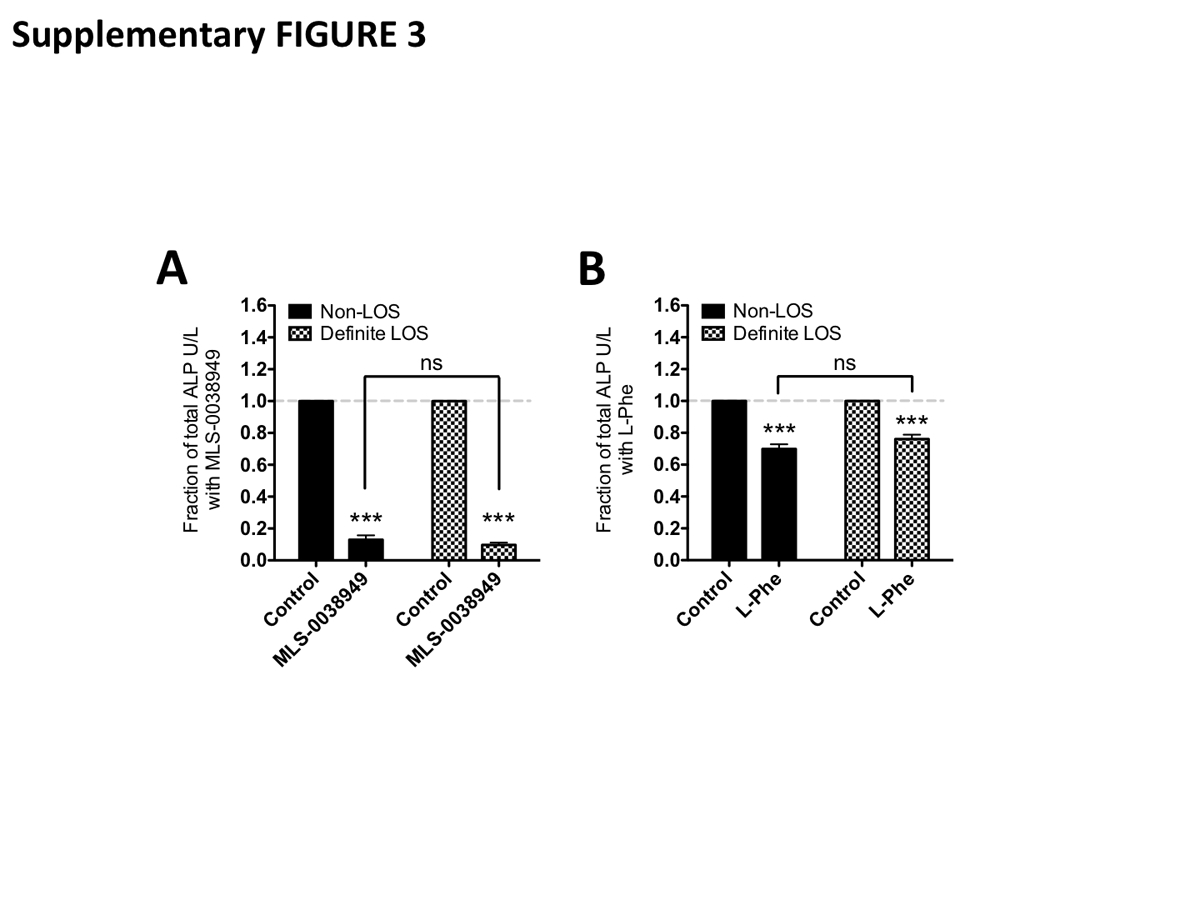

Supplement: S3 Fig — ALP activity measured in the presence of (A) MLS-0038949, a potent and selective TNAP inhibitor, or (B) L-phenylalanine, an inhibitor of IAP, PLAP, and GCAP, but a weak inhibitor of TNAP. N = 16 each group, from definite-LOS samples with sufficient volume remaining at the 4 week collection and 16 randomly selected non-LOS samples, values shown are relative to each sample’s control condition. Both inhibitors significantly reduce ALP activity, paired Student’s t-tests, *** p<0.001. Two-tailed Student’s t-tests indicate no significant differences between definite LOS and non-LOS groups for either pharmacological inhibitor. (TIF) [file pone.0175936.s003.tif]
